# Apoptosis: A Target for Anticancer Therapy

**DOI:** 10.3390/ijms19020448

**Published:** 2018-02-02

**Authors:** Claire M. Pfeffer, Amareshwar T. K. Singh

**Affiliations:** Department of Biology, Division of Natural and Social Sciences, Carthage College, Kenosha, WI 53140, USA; cpfeffer@carthage.edu

**Keywords:** apoptosis, anticancer therapy, curcumin, apoptotic evasion

## Abstract

Apoptosis, the cell’s natural mechanism for death, is a promising target for anticancer therapy. Both the intrinsic and extrinsic pathways use caspases to carry out apoptosis through the cleavage of hundreds of proteins. In cancer, the apoptotic pathway is typically inhibited through a wide variety of means including overexpression of antiapoptotic proteins and under-expression of proapoptotic proteins. Many of these changes cause intrinsic resistance to the most common anticancer therapy, chemotherapy. Promising new anticancer therapies are plant-derived compounds that exhibit anticancer activity through activating the apoptotic pathway.

## 1. Introduction

Apoptosis is the cell’s natural mechanism for programed cell death. It is particularly critical in long-lived mammals [1] as it plays a critical role in development as well as homeostasis [2]. It serves to eliminate any unnecessary or unwanted cells and is a highly regulated process. There are a wide variety of conditions that will result in the apoptotic pathway becoming activated including DNA damage or uncontrolled proliferation [3]. The apoptotic pathway is activated by both intracellular and extracellular signals. There are two different pathways that lead to apoptosis: the intrinsic and extrinsic pathways that correlate with the signal type. They are also referred to as the mitochondrial and death receptor pathways, respectively. The intracellular signals include DNA damage, growth factor deprivation and cytokine deprivation [4], whereas the most common extracellular signals are death-inducing signals produced by cytotoxic T cells from the immune system in response to cells that are damaged or infected [4]. The pathways converge at the executioner caspases. 

As soon as apoptosis is signaled, changes start to occur within the cell. These changes include activation of caspases which cleave cellular components required for normal cellular function such cytoskeletal and nuclear proteins. As a result of caspase activity, apoptotic cells begin to shrink and undergo plasma membrane changes that signal the macrophage response [2].

Apoptosis is carried out by caspases (cysteine aspartyl-specific proteases) which are a class of cysteine proteins that cleave target proteins [4]. The caspase protease activity is essential to successful apoptosis as they cleave hundreds of various proteins [3]. There are four initiator caspases (caspase-2, -8, -9, 10) and three executioner caspases (caspase-3, -6, -7) [4]. The executioner caspases cleave the target proteins that eventually leads to the death of the cell. The pathways are highly regulated so that apoptosis will only occur if signaled. The intrinsic pathway, in particular, is regulated by the B-cell lymphoma-2 (BCL-2) protein family which include proapoptotic effector proteins, proapoptotic BH3-only proteins, and antiapoptotic BCL-2 proteins [3]. The antiapoptotic BCL-2 proteins inhibit apoptosis through the inhibition of the proapoptotic BCL-2 proteins, BCL-2-associated X protein (BAX) and BCL-2 homologous antagonist killer (BAK) [4]. BH3-only proteins inhibit the antiapoptotic BCL-2 proteins. 

The dysregulation of apoptosis is a symptom in a wide variety of diseases. Accelerated apoptosis is found in infertility, immunodeficiency, and acute and chronic degenerative diseases [2], and delayed or inhibited apoptosis is present in cancer and autoimmunity. 

## 2. Apoptosis in Cancer

The hallmarks of cancer are present in all cancer cells regardless of the cause or type; these include uncontrolled growth, angiogenesis and apoptosis evasion [5,6]. The prevention of cancer is one of the main functions of apoptosis [3]. Typically, it is the intrinsic pathway that is inhibited in cancer, however, there are a wide range of means to inhibit apoptosis. The loss of apoptotic control allows cancer cells to survive longer and gives more time for the accumulation of mutations which can increase invasiveness during tumor progression, stimulate angiogenesis, deregulate cell proliferation and interfere with differentiation [2].

There are many ways in which cancer cells which evade apoptosis: caspase function can be inhibited or the trigger for apoptosis can be disabled [3]. The upregulation of antiapoptotic BCL-2 proteins and loss of BAX and/or BAK are the predominant methods of evasion. *BCL-2* is not considered an oncogene, but mutations in it enhance tumor onset [3]. The overexpression of BCL-2 protein is present in over half of all cancers, regardless of type [3]. This results in tumor cells that are resistant to any intrinsic apoptotic stimuli which includes some anticancer drugs [2].

## 3. Apoptosis and Cancer Therapy

One way of treating cancer is to gain control or possibly terminate the uncontrolled growth of cancer cells. Using the cell’s own mechanism for death is a highly effective method. Additionally, targeting apoptosis is the most successful non-surgical treatment. Targeting apoptosis is also effective for all types of cancer, as apoptosis evasion is a hallmark of cancer and is nonspecific to the cause or type of the cancer. There are many anticancer drugs that target various stages in both the intrinsic and extrinsic pathways [7,8,9]. Two common strategies for therapeutic targeting are stimulation of proapoptotic molecules and inhibition of antiapoptotic molecules [2]. Some of the targets that have been researched include ligands for death-receptors [3], inhibitors for BCL-2 [4], XIAP inhibition [3] and alkylphospholipid analogs (APL) which act as apoptotic signals [8]. Any stage in the pathways can be targeted for treatment, however, there is no indication of which target is most effective. As more apoptosis-inducing anticancer drugs are designed, the most effective targets will be determined. 

## 4. Intrinsic Pathway

**Figure 1:**
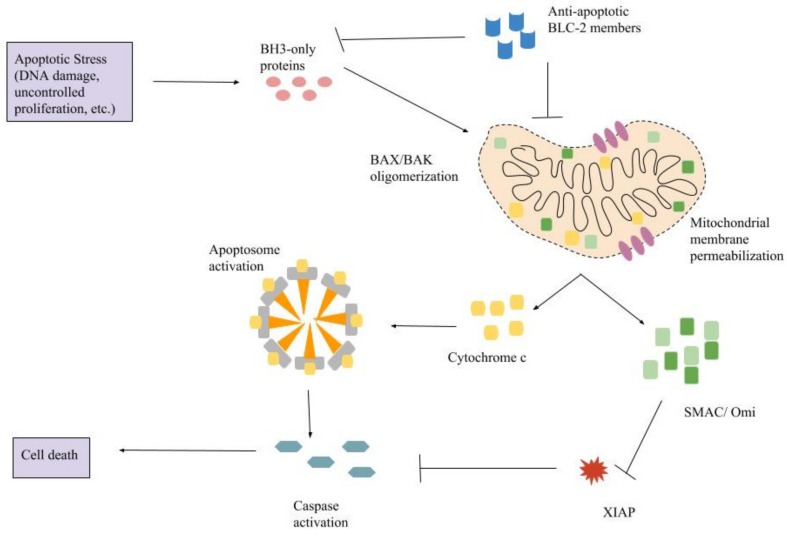
The pathway of intrinsic apoptosis. BH3-only proteins are upregulated in response to apoptotic stress. They activate BAX (BCL-2-associated X protein) and BAK (BCL-2 homologous antagonist killer) which oligomerize and results in mitochondrial membrane permeabilization. Cytochrome c, SMAC (second mitochondria-derived activator of caspase), and Omi are released and the apoptosome is formed from procaspase-9, dATP, cytochrome c, and APAF-1. Caspases are then activated and begin to cleave cellular proteins resulting in apoptosis. Arrows represent activation and T bars represent inhibition.

The intrinsic mechanism of apoptosis uses the mitochondria and mitochondrial proteins (Figure 1). Cells with damaged DNA or upregulated oncogenes can stimulate this pathway [6]. Additional stimuli for this pathway includes growth factor deprivation, surplus Ca^2+^, DNA-damaging molecules, oxidants and microtubule targeting drugs [2]. The overall pathway is regulated by the BCL-2 family of proteins [4]. Various apoptotic stimuli result in the upregulation of BH3-only proteins, which then activate both BAX and BAK [10]. BAX is regulated by p53 [11], a tumor suppressor gene. Once activated, BAX and BAK oligomerize, which leads to mitochondrial outer membrane permeabilization (MOMP). MOMP is the defining event of intrinsic apoptosis and is considered the point of no return [3]. The permeabilization allows the release of intermembrane proteins like cytochrome c, second mitochondria-derived activator of caspase (SMAC) and Omi. Upon the release of cytochrome c, the apoptosome is formed from cytochrome c, apoptotic protease-activating factor-1 (APAF-1), dATP and procaspase-9 [2]. Within the apoptosome, procaspase-9 is converted into caspase-9 [4] which activates the executioner caspases-3 and -7 [12]. The executioner caspases quickly begin to break down proteins leading to cell death. 

There are additional steps to intrinsic apoptosis that ensure cell death. Omi inhibits X-linked inhibitor of apoptosis protein (XIAP) which is an endogenous inhibitor of caspase function [3]. SMAC is released during apoptosis to inhibit inhibitor of apoptosis proteins (IAP) so that apoptosis proceeds once the apoptosome is formed [4]. MOMP will also lead to cell death if caspases are not activated. The permeabilization of the membrane leads to loss of mitochondrial function which leads to cell death [3]. There are a few cells that can survive MOMP such as neurons. It has also been found that some cancer cells are able to elude death even after MOMP [3].

## 5. Extrinsic Pathway

**Figure 2:**
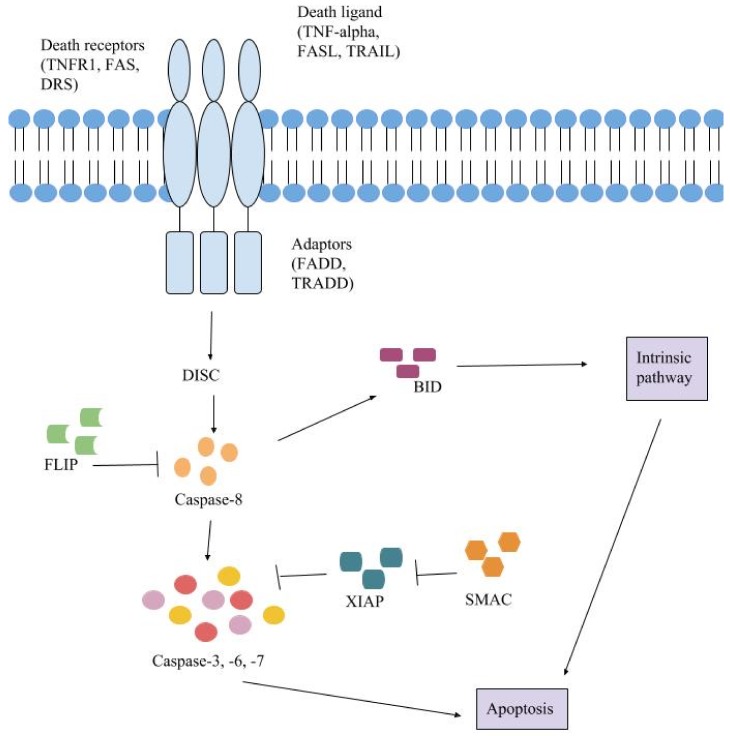
The extrinsic pathway begins with a death ligand docking on a death receptor. An adaptor protein binds to the receptor. DISC (death-inducing signaling complex) is formed from the adaptor protein and procaspases-8 and -10. Caspase-8 becomes activated which activates caspases-3, -6 and -7 and BID (BH3 interacting-domain death agonist). BID goes on to activate BAX and BAK which activates the intrinsic pathway. Caspases-3, -6 and -7 are the executioner caspases that result in cell death. Arrows represent activation and T bars represent inhibition.

The extrinsic pathway uses extracellular signals to induce apoptosis (Figure 2). Cell death signals, also known as death ligands, bind to tumor necrosis factor (TNF) family death receptors [4]. Some death ligands include Fas ligand (Fas-L), TNF-related apoptosis-inducing ligand (TRAIL) and tumor necrosis factor (TNF) [13]. An adaptor protein is recruited to the death receptor [4,14]; adaptor proteins include Fas-associated death domain (FADD) and TNF receptor-associated death domain (TRADD) [13]. Initiator procaspases-8 and -10 bind to the adaptor protein, forming the death-inducing signaling complex (DISC) [4,14]. The procaspases have a death effector domain (DED) that binds to the adaptor protein at its DED [13]. Procaspases-8 and -10 are activated by DISC. Executioner caspases-3, -6 and -7 are then activated and begin the cleavage of proteins and the cytoskeleton leading to cell death. DISC is regulated by the inhibitor, c-FLIP, which is homologous to caspase-8 yet lacks caspase activity [4].

The extrinsic and intrinsic pathways converge after the activation of caspase-8. In the extrinsic pathway, the activation of caspase-8 leads to the activation of BH3 interacting-domain death agonist (BID), a BH3-only protein [12]. BID then activates and oligomerizes BAX and BAK and the intrinsic apoptotic pathway continues. This results in both pathways to continue to propagate through their typical course ensuring that apoptosis will occur.

## 6. Apoptotic Changes in Cancer

Cancer cells evade apoptosis through a variety of mechanisms. Deviation from the normal pathways can either cause prosurvival regulation or proapoptotic regulation. While not classified as such, prosurvival genes are potentially oncogenic and can have mutations that increase their expression [15]. On this same note, proapoptotic genes may act as tumor suppressors. All of the inhibitors and activators have been found outside their normal range of expression in cancer cell lines. For instance, in almost half of all human cancers, BCL-2 expression is elevated [16].

The vast majority of traditional anticancer drugs depend on BCL-2/BAX-dependent mechanisms to kill cancer cells [16]. This leads to the failure of drugs if this mechanism is disrupted or changed in any way and forms an intrinsic chemoresistance. Additionally, the threshold for chemotherapy or radiotherapy is raised due to apoptosis defects [2] which leads to resistance to those therapies. Altered apoptotic signaling pathways promote resistance to the immune system as the immune system depends on apoptosis [2]. 

## 7. Proapoptotic Regulation in Tumor Cells

There are many signals that can occur in cancer cells that quickly lead to apoptosis despite their typical evasion of apoptosis. Cancer cells are ‘primed for death’ meaning that they are closer to triggering the apoptotic pathway than normal cells [17]. The sensitivity for apoptotic signals increases in these primed cells [3]. Priming is due to the dual upregulation of proapoptotic and antiapoptotic proteins [17], which results in cells that undergo apoptosis more quickly and easily. If upregulation of the antiapoptotic proteins is halted or disrupted, then the proapoptotic proteins can trigger apoptosis. Targeting primed cells with an inhibitor of antiapoptotic proteins could result in apoptosis and the death of the tumor cell. 

Additionally, cancer cells are more sensitive to apoptosis because of environmental stressors so that they endure low availability of nutrients or hypoxia [3]. In general, tumor cells are more sensitive to the extrinsic pathway than the intrinsic one [1], which indicates that the extrinsic pathway should be targeted for cancer therapy. Other oncogenes and tumor suppressors mediate or affect apoptosis which could be the cause for apoptotic evasion. The tumor suppressor, p53, activates transcription of proapoptotic proteins from the BCL-2 family [17]. If a tumor suppressor mutation is the cause of apoptotic evasion, then the apoptotic pathway would need to be activated in another manner. This knowledge can help predict the best and most effective mechanism to target in cancer therapy.

## 8. Prosurvival Regulation in Tumor Cells

There are numerous inhibitors of both the apoptotic pathways that are overexpressed in tumors [14]. Increased expression of antiapoptotic proteins like BCL-2 and down regulation of proapoptotic proteins like BAX are two methods for cells to resist apoptosis [2]. The defects in the apoptosis allow tumor cells to resist traditional therapies such as chemotherapy and radiotherapy. This is done by raising the threshold needed for cell death [2]. Additionally, this resistance to apoptosis can also promote resistance to the overall immune system as the immune system depends on the integrity of the apoptosis pathways [2]. 

The prosurvival proteins throughout the apoptotic pathway include BCL-2, BCL-xl, BCL-w, mcl-1, A1, NR-13, BHRF1, LMW5-HL, ORF16, KS-BCL-2 and E1b-19K [15]. Many of these proteins have been found overexpressed in cancer. For example, BCL-2 has elevated gene expression in over half of all cancers [16] and XIAP is overexpressed in many different tumors [17]. The overexpression of these antiapoptotic proteins inhibits apoptosis from a variety of signals including hypoxia, growth factor deprivation and oxidative stress [16]. 

While prosurvival proteins are overexpressed, the proapoptotic proteins are under-expressed or inhibited. Caspases, particularly the executioner caspases, are under-expressed in tumor cells [17]. Deletions or inactivation mutations in caspase genes have been observed in various cancers [16]. Neuroglobin (NGB), a globin found preferentially in neurons, associates with cytochrome c [18]. This impairs its release into the cytosol and activation of the apoptosome. NGB has been found at higher concentrations in cancer cells and is believed to render cells insensitive to chemo- and radiotherapy due to its interference in the intrinsic apoptotic pathway [19]. Additionally, cancer cells have found ways to survive past MOMP. In order for an apoptotic response to occur, 15% of the cell’s mitochondrial population must undergo MOMP [17]. If cells are able to halt MOMP before 15% of the mitochondria are permeabilized, then the apoptotic response will not continue to propagate. This gives cancer cells more time to prevent a full apoptotic response. 

## 9. Blebbishield Formation

One mechanism that cancer stem cells use to evade apoptosis is the formation of blebbishields. The emergency program is activated in order to save apoptotic cancer stem cells [20]. The apoptotic blebs fuse together to form a structured sphere termed blebbishields [21]. Blebbishield activation has been linked to immune evasion [22], apoptosis evasion [21], tumorigenesis [22], enhanced glycolysis [23], generation of chromosomal instability [22], drug resistance [20] and metastasis [22]. Cells undergoing blebbishield formation show visual signs of apoptosis, but that response is halted and results in the cell survival [21]. Blebbishields use a similar mechanism found in mitosis; cells undergoing blebbishield formation resemble mitotic cells rather than apoptotic cells [20]. Endocytosis [20] and endocytosis-driven serpentine filopodia formation [24] are active in blebbishields to prevent the full apoptotic response. Apoptosis typically culminates in secondary necrosis due to a lack of ATP; blebbishields are able to avoid secondary necrosis through activation of glycolysis [23].

Caspases [21], BAD activation [24] and K-ras signaling [23] have all been found to play a role in the formation of blebbishields. K-ras signaling regulates the phosphorylation of BAD [23], activating BAD which goes on to regulate glycolysis. BAX and BAK also boost glycolysis [23], providing the forming blebbishield with enough ATP to avoid secondary necrosis. Additionally, BAX p18 fragment has been found to play a major role in the signaling of MOMP and is found at low levels during blebbishield formation [23]. Overall, during blebbishield formation, the mitochondria are protected to ensure that adequate levels of ATP are produced and secondary necrosis is avoided [22].

Survival of cancer stem cells must be blocked concurrent with therapies targeting apoptosis to ensure therapeutic success. A number of potential candidates have been found including caspase inhibitors [21], Smac mimetics [23] and internal ribosome entry site (IRES) translation inhibitors [24]. Antiapoptotic proteins such as cIAP-2 and XIAP are under the control of IRES translation [22]. IRES translation promotes survival through translation of cIAP-2 which ignites the process [24] and shifts the antiapoptotic versus proapoptotic balance towards antiapoptotic resulting in cell survival [22]. Inhibiting IRES translation would prevent initiation of the blebbishield formation. N-Myc is an IRES-translational target that has been targeted to prevent blebbishield formation [24].

## 10. Plant-Derived Compounds Exhibiting Anti-Cancerous Activity

In light of increasing resistance to chemo- and radiotherapy along with the toxicity of these traditional therapies, a new, non-toxic anticancer treatment is needed. Additionally, chemotherapeutic agents are toxic to both normal cells and tumor cells. The optimal therapy would be to be able to differentiate between the cell types. Plant-based or -derived compounds are typically non-toxic to normal cells. For over 5000 years, plants have been utilized as medicines and therapies; a quarter of all modern medicine is directly or indirectly derived from plants [25].

Graviola is a fruit tree that has been used in both alternative and traditional medicine for a wide variety of aliments [26], particularly for its anticancer properties. It has been shown to inhibit BCL-2 proteins while increasing BAX and promoting apoptosis [27]. The mechanism graviola uses is still unknown, however, it is a potential anticancer treatment due to its nontoxicity towards healthy cells [26]. This makes graviola an exciting new possible therapy especially in comparison to the current treatments of chemo- and radiotherapy.

There are many other plant-derived compounds that induce apoptosis in cancer cells (Table 1). These include black cohosh of *Actaea racemosa* [28], Juglone from *Juglans mandshurica* [29] and genistein [30]. Quercetin, which is found in apples and red onions, activates caspases which leads to the apoptotic response [30]. Green tea is also thought to induce apoptosis in cancer cells, specifically through the compound epigallocatechin-3-gallate [31]. Aloe-emodin, found in *Rheum palmatum*, also has caspase-activation activity in cancer cells [31]. All of these plant-derived compounds have yet to be fully investigated but are noteworthy compounds that could be the future of cancer therapy. The nontoxicity towards normal, healthy cells while still attacking and producing an apoptotic response in cancer cells make these compounds particularly intriguing. 

Curcumin, a polyphenolic compound found in turmeric, is derived from the rhizomes of *Curcumin longa* [38]. Turmeric powder contains about 77% curcumin [39]. Curcumin has been found to have a wide range of properties including anti-inflammatory, anti-oxidant and anti-carcinogenic, among others [39]. Turmeric has been used for thousands of years and has been frequently cited in both traditional Chinese and Ayurveda medicine [39]. 

The use of curcumin has been approved by both the Food and Drug Administration and the World Health Organization [40]. It is also abundantly used as a food coloring [38] indicating its nontoxicity. Up to 12,000 mg/day of curcumin is both tolerable and safe [41]. When compared to cytotoxic drugs, the toxicity levels of curcumin are minimal. Curcumin is not only nontoxic, but it is widely available and affordable [33,38]. The most attractive quality of curcumin is that it is toxic to cancer cells, yet is cytoprotective to normal cells [41]. 

Curcumin’s use has been limited by low solubility, rapid metabolism, poor bioavailability, low bioactive absorption and low targeting efficacy [38], among others. In order to enhance its effectiveness, curcumin can be paired with other anticancer drugs like 5-fluorouracil, oxaliplatin, or gemcitabine [40]. Various delivery methods have been developed for curcumin including nanoparticles, powder and capsules [39]. We have shown earlier [42,43] that a formulation of curcumin into nanoparticles, called curcumin-ND (curcumin nanodisks), enhanced the biological effects of curcumin on apoptosis via reactive oxygen species generation and activation of the caspase-3 pathway, as well as cell cycle arrest at the G1-S phase [42]. The curcumin-ND increased growth arrest at G1 correlated with a decrease in cyclin D1 levels in Mantle cell lymphoma cells [42]. The formulation of curcumin into nanoparticles (termed nanodisks, ND) are water soluble and have therapeutic advantages such as enhanced payload delivery, nanoscale size, high curcumin binding capacity and targeting potential [44].

People from southwest Asia, who regularly consume turmeric in their diet, have the lowest incidence of most cancer types [33]. This correlation could be an indicator of the anticancer activity of curcumin. 

There are multiple signaling pathways that curcumin has the ability to influence including cell proliferation, cell survival, caspase activation, death receptor, mitochondrial, protein kinase and tumor suppressor pathways [33]. Through these interactions, curcumin has been found to suppress tumor cells during initiation, progression and metastasis [39]. Curcumin’s multiple pathway interaction can be explained by its ability to physically bind with 33 different proteins [33]. The actual mechanism that makes curcumin potent to a wide variety of cancer cells is still unknown, but further research is needed. The effect that curcumin has on cancer cells is universal [39] to all cancer types and has been found to be effective and successful in breast, lung, prostate, pancreatic, oral, colorectal, multiple myeloma and head and neck squamous cell carcinoma.

Curcumin interacts with both apoptotic pathways. It is able to interfere at many different points in the signaling cascade. BCL-2 and XIAP are inhibited by curcumin [33] which leads to increased expression of BAX and BAK. Curcumin also increases the ability for mitochondria to undergo mitochondrial membrane permeability [33] leading to increased release of cytochrome c which causes caspase activation and the apoptotic response. 

Graviola, curcumin and other plant-derived compounds have all presented evidence of their anticancerous activity, particularly through the apoptotic pathways. With the increase in intrinsic chemoresistance and toxicity of current treatments, these plant-derived compounds are possible nontoxic anticancer therapies.

## 11. Conclusions

Targeting the apoptotic pathway is an intriguing approach to finding new anticancer therapies as it is nonspecific to cancer type. There are numerous mutations found in both extrinsic and intrinsic pathways in cancer, allowing the cells to evade apoptosis which is a hallmark of cancer. The ability to target and activate an apoptotic pathway would provide a more universal cancer therapy. Particularly promising compounds to trigger apoptosis are many plant-derived compounds that are additionally nontoxic to healthy cells. 

## Figures and Tables

**Table 1:** Summary of plant-derived compounds and their mechanism for inducing apoptosis.

Compound	Found in	Mechanism
Aloe-emodin	*Rheum palmatum*	Induces cytochrome c release [32]
Black cohosh	*Actaea racemosa*	Activates caspases [28]
Curcumin	Tumeric	Inhibits BCL-2 and XIAP [33]
Epigallocatechin-3-gallate	Green tea component	Activates cell death receptors [34]
Genistein	Soybeans	Cell cycle arrest activation [35]
Graviola	*Annona muricata*	Inhibits BCL-2 and activates BAX [26]
Juglone	*Juglans mandshurica*	Increase caspase 9 cleavage [36]
Quercetin	Bark of many plants	Modulating cell cycle regulators to arrest the cell cycle [37]
